# Neuromorphic Devices and Computing for Sensing, Memory, and Control

**DOI:** 10.1002/advs.77116

**Published:** 2026-08-13

**Authors:** Zhengguang Zhu, Nicholas Schaffer, Xiao Yang

**Affiliations:** ^1^ Department of Biomedical Engineering Johns Hopkins University Baltimore Maryland USA; ^2^ Department of Electrical and Computer Engineering Johns Hopkins University Baltimore Maryland USA; ^3^ Department of Materials Science and Engineering Johns Hopkins University Baltimore Maryland USA; ^4^ Center for MicroPhysiological Systems Johns Hopkins University Baltimore Maryland USA; ^5^ Translational Therapeutics and Regenerative Engineering Center Johns Hopkins University Baltimore Maryland USA

**Keywords:** artificial neural network, biological neural network, bioelectronics, brain–computer interface, neuromorphic engineering, neuromorphic hardware, organoid

## Abstract

Neuromorphic devices are bioinspired electronic systems that mimic key structures and functions of the nervous system, enabling integration and communication between living tissues and machines. This review examines how neuromorphic devices and computing are designed to emulate the structure, organization, and function of the nervous system. For neuromorphic devices, we first describe strategies that mimic subcellular neural functions. We then highlight how device architecture recapitulates biological topology from subcellular components to brain networks. We next summarize how neuromorphic devices emulate sensory and sensorimotor (sensory–modulation) neural circuits. For neuromorphic computing, we review recent advances in artificial and biological neuromorphic computing, including spiking neural networks, bioinspired learning algorithms, and applications. We also discuss emerging biohybrid intelligence systems leveraging two‐ and three‐dimensional biological networks as computational units. Finally, we outline key challenges, potential milestones, and future directions.

## Graphical abstract

This review introduces neuromorphic devices made from diverse materials. These devices mimic neuronal functions and architectures and, when integrated with artificial or biological computing, can form closed loops with neurons for pressure, optical, acoustic, and biochemical sensing and modulation.

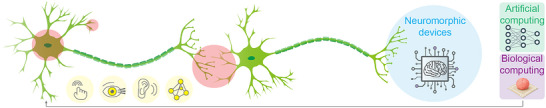

## 1 Introduction

The nervous system is a complex system that performs processes including perception, computation, learning, and memory through parallel, event‐driven interactions mediated by ionic and electronic signaling. Conventional von Neumann computers separate processing and memory units, requiring constant data transfer between them. This transfer increases latency [1] and leads to greater energy inefficiency compared with the human brain [2]. In contrast, the brain stores information locally at the sites where it is processed: neurons and synapses [3]. Neurons are interconnected via synapses, and changes in synaptic strength provide the mechanisms for learning and memory. Thus, systems that leverage these biological principles can be orders of magnitude more effective than conventional digital methods [1]. The functions of subcellular components, including the soma, dendrites, and synapses, are integrated to support higher‐order neural architectures such as dendritic trees, neurons, and interconnected neural networks. The organization of these neural networks across the central and peripheral nervous system gives rise to larger neural circuits throughout the body. The mechanisms of the nervous system have inspired the development of neuromorphic devices, which aim to mimic key features of neural information processing in physical hardware to overcome the limitations of traditional devices.

Neuromorphic computing differs fundamentally from von Neumann architecture in its information retrieval and processing strategies. Inspired by theoretical and experimental neuroscience, these algorithms are designed to emulate the computational mechanisms of the brain. Rather than abstracting information storage and retrieval into separate components, neuromorphic approaches integrate them within the same framework. Information in neuromorphic computing is represented as discrete spikes, enabling event‐driven parallel processing instead of sequential execution. The integration of storage and computation allows for efficient processing far exceeding traditional architectures [3, 4].

**FIGURE 1:**
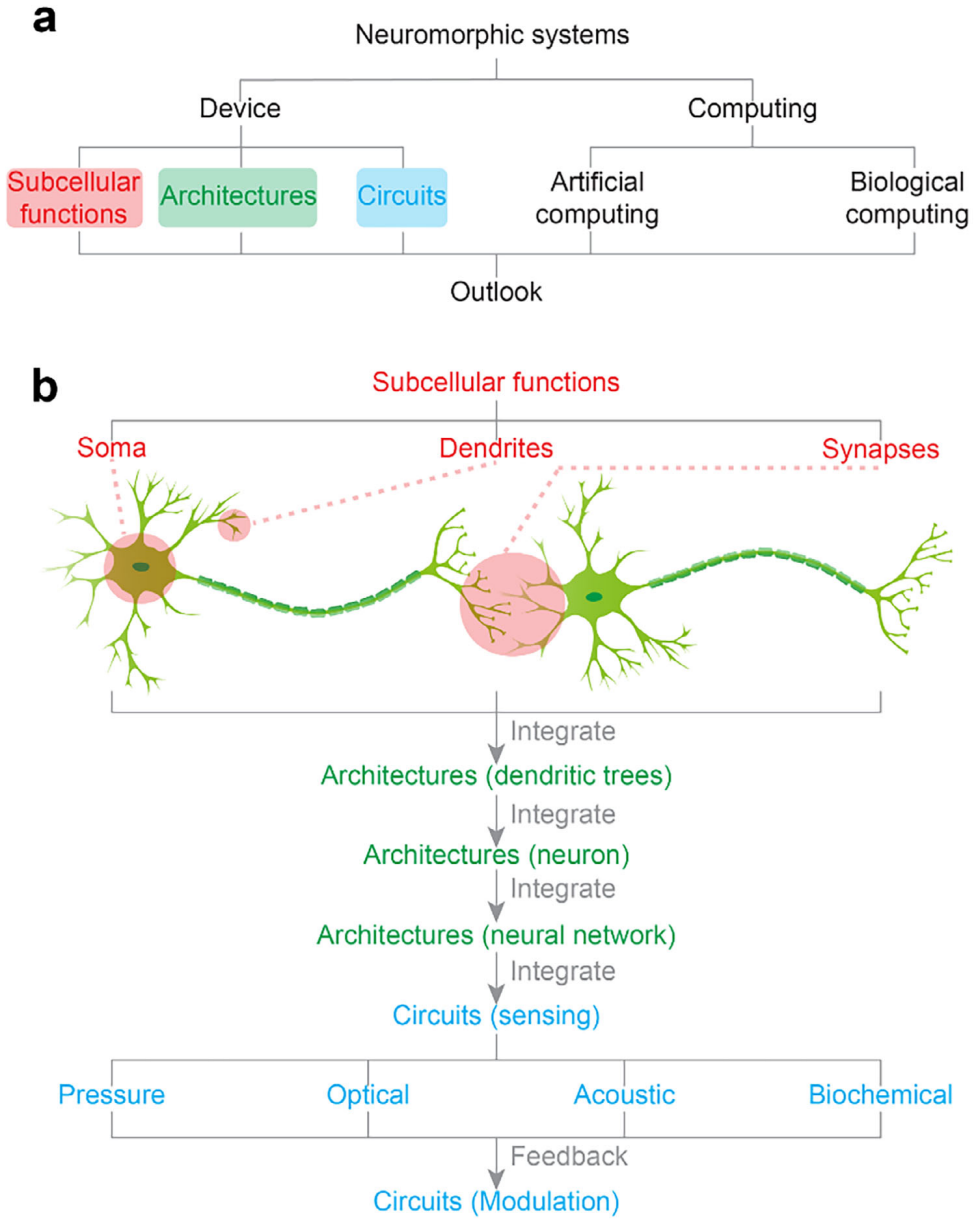
Overview of the article framework. (a), Organization of the article, covering neuromorphic devices, and neuromorphic computing. (b), Diagram illustrating the connections among neuromorphic devices that mimic subcellular functions, neural architectures, and neural circuits.

In this review, we discuss recent advances in neuromorphic devices and computing (Figure 1). We categorize neuromorphic devices into three levels: neural function‐mimicking, neural architecture‐mimicking, and neural circuit‐mimicking, and explain how each mimics specific aspects of the biological nervous system. We then review computational developments, including bioinspired learning algorithms and architecture for spiking neural networks implemented on both conventional and neuromorphic hardware. Importantly, we highlight applications of neuromorphic computing that offer efficiency advantages over traditional systems. We also cover emerging biohybrid strategies that leverage biological neural networks as computing hardware. Finally, we outline future directions, key challenges, and the need for further integration of neuromorphic devices and computing.

## 2 Neuromorphic Devices Mimicking Subcellular Neural Functions

The nervous system exhibits activity‐dependent synaptic plasticity, in which neurons dynamically change their synaptic strengths based on past experiences [5]. Postsynaptic neurons in the brain integrate spike trains from presynaptic neurons to generate action potentials [6]. The soma initiates spikes which propagate through the nervous system [7]. Dendrites integrate and filter postsynaptic signals [8]. Synapses transform information between neurons [9]. Neural function‐mimicking neuromorphic devices aim to recapitulate specific neural functions such as synaptic plasticity, signal integration, spike generation, and temporal filtering. Recent advances have focused on mimicking the functions of neuronal soma, dendrites, and synapses using a range of systems and materials. Importantly, neuromorphic devices that mimic different subcellular neural functions can also be integrated to form neuron‐like functional units, such as by combining memristors, transistors, and control resistors to couple dendrite‐like signals with soma‐like thresholding and spike generation [10]. In this section, we discuss the function of each subcellular component*—*soma, dendrites, and synapses*—*and explain how neuromorphic devices emulate their key functions (Table 1).

**TABLE 1:** Comparison of materials for neuromorphic devices.

	Metal oxides	Solid electrolytes	Organic materials	Phase‐change materials	Ferroelectric materials
Operational principles	MOSFET	Ion mobility under voltage	Memristor	Structural phase transitions	Electrically switchable polarization
Advantages	High mobility [6], scalable fabrication [7], low off‐current [8], multi‐terminal control [9, 10]	Solid‐state stability [11], strong electric double‐layer coupling [12], short‐term memory [13]	Long‐term plasticity	Nonvolatile conductance storage [14], synaptic weight update	Nonvolatile conductance [15], low‐power operation [16]
Limitations	Threshold‐voltage instability [17], oxygen defect control difficulty [17]	Speed and bandwidth limits [18, 19], volatility [20]	Poor stability [21], conductance state noise [21]	Asymmetric phase transitions [22], nonlinear conductance update [2], conductance drift [22]	Wake‐up effect [23], fatigue [23], leakage current increase [23]
Applications	Neuromorphic soma	Neuromorphic dendrites	Neuromorphic synapses	Neuromorphic synapses	Neuromorphic synapses

### 2.1 Neuromorphic Soma

The soma is the main body of a neuron that integrates transient synaptic inputs from dendrites and contributes to spike initiation and propagation in the nervous system [24]. Dendritic Ca^2+^ spikes can trigger a burst of action potentials in the soma [25]. The combination of input integration and thresholding determines if an action potential is generated.

To emulate somatic input integration and thresholding, researchers have employed the metal‐oxide‐semiconductor field‐effect transistor (MOSFET), where the operation is governed by capacitive charge accumulation and nonlinear activation. A MOSFET consists of a gate electrode insulated from a semiconductor channel by a thin oxide layer. A source region and a drain region are formed by doped semiconductors which are separated by a substrate. For instance, in an n‐type MOSFET, n‐type source and drain regions are embedded within a p‐type substrate [26]. Applying a voltage to the gate produces an electric field across the oxide layer without direct current flow, causing charge to accumulate at the semiconductor‐oxide interface. When the gate voltage exceeds the threshold, an inversion layer forms, creating a conductive channel between the source and drain. The gate capacitance enables temporal integration of input voltages, analogous to membrane potential integration by synaptic currents [10, 11, 12]. The MOSFET can be fabricated with palladium (Pd) as the gate electrode, hafnium dioxide (HfO2) as the dielectric layer [27], and n‐type indium gallium arsenide (InGaAs) as the source and drain [28]. This gate‐controlled channel formation resembles the neuronal soma, where subthreshold leakage currents provide a natural decay mechanism analogous to the membrane leak in neurons. When the gate voltage exceeds the threshold, the drain current rises sharply, mimicking the neuron's all‐or‐none firing behavior [27, 28, 29]. The drain current also changes with the gate voltage [28]. Unlike conventional MOSFET electronics which are mainly used as deterministic switching elements, neuromorphic MOSFET electronics use threshold shift, floating‐body effect, charge trapping, subthreshold dynamics, and transient current responses to function as artificial neural elements [29].

MOSFET‐based technology is mature in fabrication and compatible with electronic circuits. Standard silicon CMOS processing provides high yield and low variability for MOSFET production [29]. However, MOSFET platforms are limited by the requirement for integrating multiple transistors per function [32], increasing device area and energy consumption. Future MOSFET‐based neuromorphic devices can be further improved through manipulation of oxygen‐vacancy order, better gate control, and improved flexibility.

### 2.2 Neuromorphic Dendrites

Neuronal dendrites express voltage‐dependent Ca^2+^ and Na^+^ channels and are responsible for integrating and filtering postsynaptic signals and performing spatiotemporal summation before signals reach the soma [34]. In pyramidal neurons, proximal dendrites receive excitatory inputs from multiple sources [25]. Dendritic spikes that successfully propagate to the soma summate, triggering an action potential once the membrane potential exceeds the threshold. Dendrites also act as filters that reduce the amplitude and slow the kinetics of synaptic inputs [35].

**FIGURE 2:**
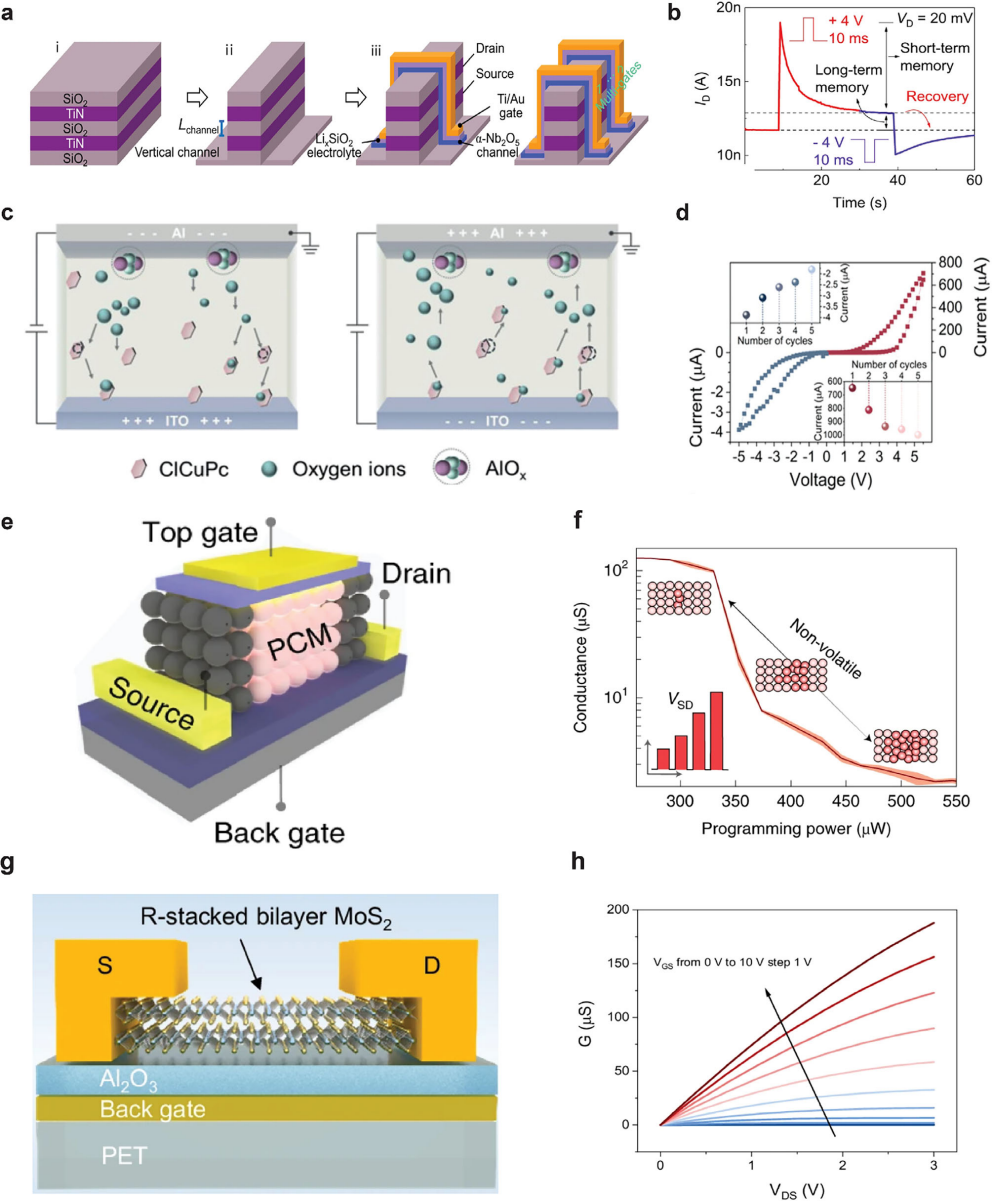
Neuromorphic devices mimicking subcellular neural functions. (a), The fabrication process and schematic of a memristor. The source and drain electrodes are in purple, the substrate is in gray, the channel is in blue, and the gate electrode is in yellow. Reproduced with permission from [30], licensed under CC‐BY. (b), The current response of the device in a showing hybrid short‐term and long‐term memory. Reproduced with permission from [30], licensed under CC‐BY. (c), Schematic diagrams showing memristive switching mechanisms. Reproduced with permission from [31], copyright 2020 Wiley‐VCH. (d), *I*–*V* curve of the memristor from c. Reproduced with permission from [31], copyright 2020 Wiley‐VCH. (e), Schematic of a device based on PCM. Reproduced with permission from [32], licensed under CC‐BY. (f), The relationship between PCM conductance and programming power from e. Reproduced with permission from [32], licensed under CC‐BY. (g), Schematic of a device based on ferroelectric material. Reproduced with permission from [33], licensed under CC‐BY‐NC‐ND. (h), The relationship between drain conductance and drain‐source voltage VDS for the device from g. Reproduced with permission from [33], licensed under CC‐BY‐NC‐ND.

To emulate dendritic integration, propagation, and spatiotemporal summation, researchers have developed dynamic oxide‐based memristors that use multiple oxide layers to provide conductance modulation similar to dendritic short‐term plasticity. A memristor is a metal‐insulator‐metal sandwich structure in which ions migrate within the switching layer to modulate device conductance. When an external voltage is applied between two electrodes, oxygen vacancies in the insulator layer drift toward the metal‐insulator interface under the electric field. The accumulation of oxygen vacancies at the interface enhances conductivity. When the voltage is removed, oxygen vacancies diffuse back due to concentration gradients, preventing the formation of a stable conductive path and suppressing long‐term memory [30, 36]. For example, titanium (Ti) and gold (Au) can be used for gate electrodes, lithium‐doped silicon dioxide can be an ion‐conducting layer, and α‐niobium pentoxide (α‐Nb_2_O_5_) can function as the memristive switching material (Figure 2a) [30]. The vertical dual‐gate neurotransistor exhibits short‐term memory behavior (Figure 2b) [30], and the vacancy drift‐diffusion process mimics temporal filtering and signal attenuation of dendrites. Compared to traditional electronics, oxide‐based memristors provide nonvolatile weight storage, higher synaptic density, and in‐memory computing capability.

Oxide‐based memristors provide a platform with a compact footprint and low energy requirements [37]. Fabrication using passive memristive crossbars offer high‐density production [38]. However, they can be limited by filamentary switching variability, causing device‐to‐device and cycle‐to‐cycle variations [37]. Despite their high‐density scalability, large memristive arrays suffer from sneak currents, voltage drops, leakage currents, yield loss, and filament‐control problems [38]. Future development of oxide‐based memristors can focus on improving switching controllability, array yield, CMOS integration, and long‐term reliability.

Beyond oxide‐based systems, solid electrolytes can also serve as the active switching media in electrolyte‐gated transistors. An applied gate voltage redistributes mobile ions in the solid electrolyte, causing them to accumulate at the electrolyte‐oxide interface, thereby modulating the channel carrier density and drain‐source conductance. After the gate voltage is removed, ions diffuse back and the conductance relaxes to baseline [39]. Compared to conventional electronics, solid electrolytes provide short‐term memory, integration of memory and processing, and biologically inspired ion dynamics [20, 40].

Solid electrolytes use mobile ions to modulate electronic conductance with low power consumption, high transconductance, and improved biocompatibility [41]. However, the speed and bandwidth of solid‐electrolyte‐based neuromorphic devices are limited by slower ion movement compared to electrons [41]. Ion movement driven by a reversed electric field or concentration gradient can limit the stability of electrolyte‐based devices. Future improvements of solid‐electrolyte‐based neuromorphic devices can focus on higher speed and bandwidth as well as long‐term stability.

Together, dynamic memristors and electrolyte‐gated transistors offer a platform for emulating dendritic computation, including temporal integration, filtering, and short‐term plasticity.

### 2.3 Neuromorphic Synapses

Neuron synapses are intercellular junctions responsible for information transfer from one neuron to another [42]. The synapse is comprised of three parts: the presynaptic terminal, the synaptic cleft, and the postsynaptic membrane. The presynaptic terminal releases neurotransmitters, including glutamate in some cases, which are stored in synaptic vesicles. Following Ca^2+^ influx, glutamate is released and diffuses into the synaptic cleft, binding to postsynaptic receptors such as *N*‐methyl‐D‐aspartate (NMDA) and α‐amino‐3‐hydroxy‐5‐methyl‐4‐isoxazole propionic acid (AMPA) receptors [42]. Synaptic plasticity includes long‐term potentiation (LTP) and long‐term depression (LTD), which are mediated by increasing and decreasing neurotransmitter receptor density on the postsynaptic membrane. Neuron synapses generally follow the Hebbian theory: “neurons that fire together, wire together.”

To emulate synaptic plasticity, researchers have developed multiple devices and materials. Memristive devices are widely used given their ability to store conductance states. A memristive device typically consists of a switching layer sandwiched between two electrodes. When a voltage is applied, the resulting electric field drives the migration of oxygen vacancies, which can form a conductive filament between two electrodes. After the voltage is removed, the conductive filament can be retained, enabling long‐term plasticity analogous to synaptic weight changes in neurons [31, 43, 44]. Device conductance can be tuned by pulse amplitude, duration, or frequency to mimic synaptic plasticity. An example memristive switching layer is the organic semiconductor chlorinated copper phthalocyanine (ClCuPc) (Figure 2c) [31]; its *I*–*V* curve demonstrates resistive switching behavior (Figure 2d) [31]. Neuromorphic synapse function can be enhanced by introducing artificial biomembranes. In an organic electrochemical transistor neuromorphic device (ENODe), 1‐palmitoyl‐2‐oleoyl‐snglycero‐3‐phosphocholine lipid with or without the addition of cholesterol and sphingomyelin is used to emulate the membrane on biological synapses [45].

The relatively simple metal‐insulator‐metal or metal‐oxide‐metal structure of memristive devices makes them compact and suitable for crossbar integration [46]. Memristive devices also have high scalability owing to the feasibility of high‐density and parallel operation of two‐terminal crossbars [38]. However, fabricating memristive devices at the array level remains complex [38]. In addition, the stability of memristive devices is limited by cycle‐to‐cycle variability, device‐to‐device variability, and state‐retention drift [47]. Future improvements can focus on analog conductance updates that enable separable conductance states, reproducible high‐yield fabrication, and lower variability.

Phase‐change materials (PCMs) provide an alternative mechanism for neuromorphic synapses based on structural phase transitions. PCMs exhibit two solid states, amorphous and crystalline, under different temperatures and with distinct electrical resistances [32]. In a PCM device, a PCM layer is sandwiched between two electrodes (Figure 2e) [32]. Electrical pulses induce Joule heating: strong pulses can heat PCMs above their melting temperature to change them into an amorphous state with a higher electrical resistance; weaker pulses heat PCMs above the crystallization temperature, allowing them to form a crystalline state with a lower electrical resistance (Figure 2f) [32, 48]; intermediate pulses can modulate the degree of crystallization to adjust PCM conductance. The phase state remains stable after power removal, mimicking long‐term memory of neurons. Compared to conventional electronics, PCM‐based devices provide nonvolatile analog conductance states and support analog in‐memory computation.

PCM‐based devices have the advantages of nonvolatility, multilevel conductance, and compatibility with computing arrays [49]. However, conductance updates in PCMs are asymmetric. The crystallization of PCM can be gradual, whereas amorphization is abrupt [2]. PCMs also suffer from conductance drift. Future development of PCM‐based devices can focus on gradual potentiation and depression, reduction of conductance drift, and CMOS integration.

Ferroelectric materials offer another route to emulate synapses through electrically switchable polarization. Many ferroelectric materials adopt a perovskite structure. Above the Curie temperature, crystals are centrosymmetrical with no net polarization; below the Curie temperature, ions shift, breaking the symmetrical structure into tetragonal [50]. Devices using ferroelectric materials typically place a ferroelectric layer between the gate electrode and the channel (Figure 2g) [33]. When an external electric field is applied, the polarization direction can be reversibly modulated, and remains stable after field removal [33, 50, 51]. The net depolarization creates an internal electric field that triggers the formation of a conductive path between source and drain in the semiconductor and thus modulates device conductance (Figure 2h) [33]. Compared to traditional electronics, ferroelectric devices provide fast polarization switching, and conductance nonvolatility.

Ferroelectric devices possess conductance nonvolatility, fast switching, low‐current operation, and potential CMOS compatibility [15]. However, ferroelectric devices are limited by wake‐up effects, fatigue, and increasing leakage current [15]. Future development of ferroelectric devices can focus on reducing synaptic update drift, fatigue, and leakage current.

## 3 Neuromorphic Devices Integrating Functional Components into Neural Architectures

In the brain, pyramidal neurons have multiple short basal dendrites and a large apical dendrite. After integrating signals from these dendrites, pyramidal neurons send signals across cortical layers; for example, layer 4 receives apical dendritic inputs from layer 5 pyramidal neurons as well as basal inputs from layer 3 pyramidal neurons [52, 53]. Architectural neuromorphic devices seek to recapitulate the spatial and organizational features of biological nervous systems. Rather than focusing solely on individual functions, they emphasize the arrangement and interaction of components within the architecture. We also address the integration of neuromorphic devices that mimic different subcellular neural functions to form a biomimic neuron. Such architectural devices bridge the gap between subcellular‐level components and system‐level neural circuits.

### 3.1 Neuromorphic Dendritic Trees

In biological neurons, dendritic trees consist of multiple branches that generate localized dendritic spikes via voltage‐dependent ion channels. These branch‐specific events enable complex integration of synaptic inputs before signals converge at the soma and axon initial segment to determine action potential initiation [25]. Emulating dendritic tree‐level computation therefore requires device architectures that go beyond single‐dendrite devices.

**FIGURE 3:**
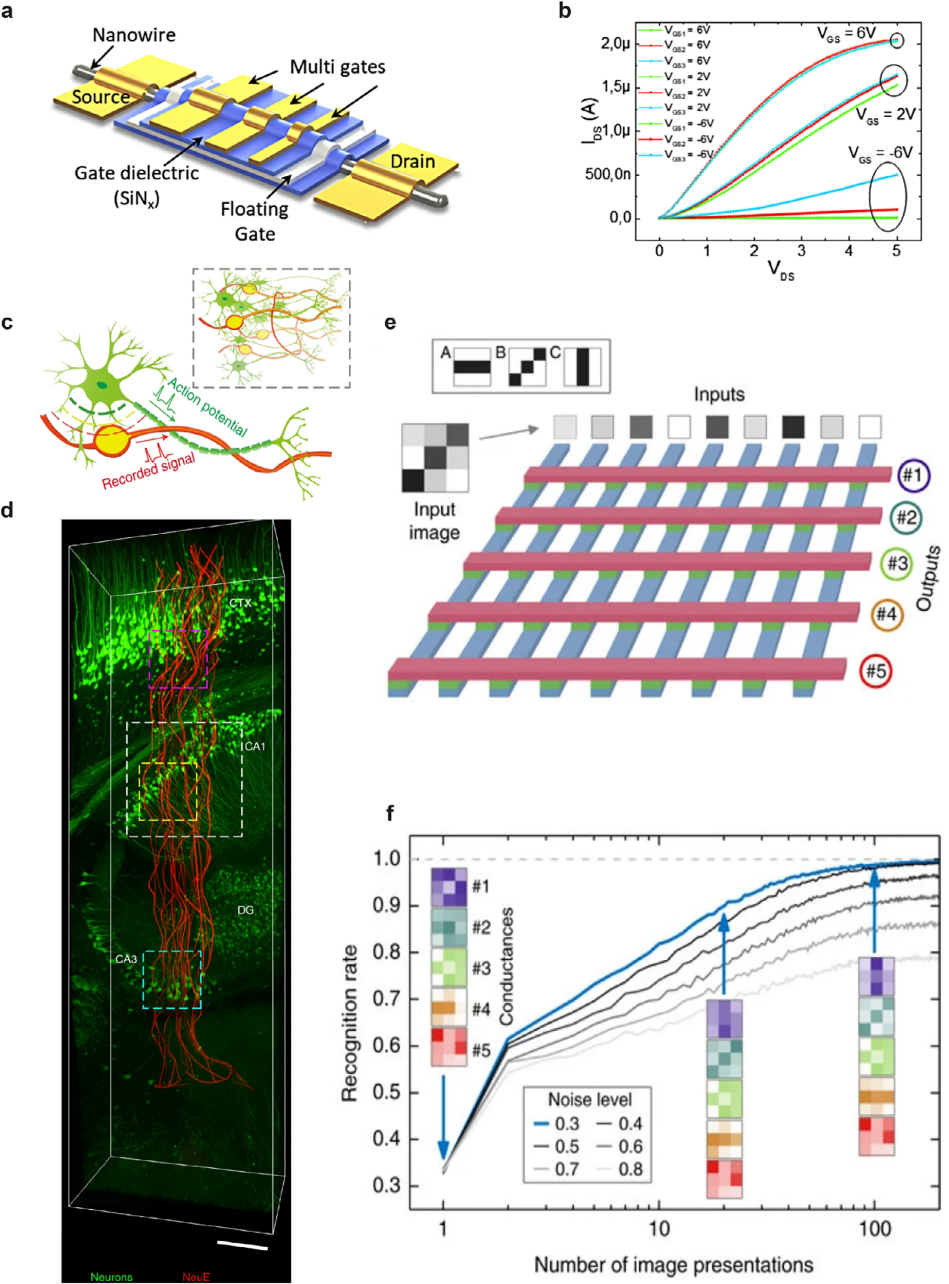
Neuromorphic devices integrating functional components into neural architectures. (a), Schematic of a multi‐gate transistor. Reproduced with permission from [10], licensed under CC‐BY. (b), Relationship between drain‐source voltage V_DS_ and the drain‐source current I_DS_ for the device in a. Reproduced with permission from [10], licensed under CC‐BY. (c), Schematic showing the architectural similarity between NeuE and neurons. Reproduced with permission from [54], copyright 2019 Springer Nature. (d), Three‐dimensional reconstruction of the interface between neurons (green) and NeuE (red). Reproduced with permission from [54], copyright 2019 Springer Nature. (e), An array of ferroelectric memristors showing nine input neurons connected to five output neurons. Reproduced with permission from [55], licensed under CC‐BY. (f), Conductance maps of memristors in each line from the array in e. Reproduced with permission from [55], licensed under CC‐BY.

To mimic dendritic trees, one approach is a multi‐gate MOSFET, in which multiple independent gates couple to a shared semiconductor channel (Figure 3a) [10]. Each gate functions as a neuromorphic dendritic branch and receives an input voltage that modulates channel carrier density [10]. The combined influence from multiple gates produces summation of inputs within the channel, analogous to signal integration across a dendritic tree. At the same drain‐source voltage V_DS_, different control gates can induce different drain‐source currents I_DS_, and a single control gate can induce different I_DS_ (Figure 3b) under different voltages [10]. Such mechanisms mimic key aspects of dendritic responses.

By integrating multiple gates, MOSFETs can mimic dendritic branches; however, multiple gates also introduce gate‐to‐gate crosstalk. In addition, current multi‐gate MOSFETs face challenges in replicating real dendrites that possess more complex branching morphology, active ion channels, local spikes, and compartmental plasticity. Future development of multi‐gate MOSFETs should further address biological fidelity.

### 3.2 Neuromorphic Neurons

A biological neuron consists of three primary components: dendrites, soma, and axon. Pyramidal neurons, one of the most prevalent neuron types in the cerebral cortex, have a teardrop‐ or pyramid‐shaped soma, a spray of dendrites, and a long projecting axon responsible for signal transmission [56]. Neurons are mechanically soft: for hippocampal neurons, the soma has a Young's modulus of 0.7 ± 0.2 kPa, dendrites 2.5 ± 0.7 kPa, and axons 4.6 ± 1.5 kPa [57]. Some axons are ensheathed by myelin, a multilayered membrane sheath that defines nodes of Ranvier between long axon segments [58]. Myelination reduces the energy consumption of restoring ion gradients via Na+/K+‐ATPase and increases signal conduction velocity [58].

To emulate the soma and myelinated axons of neurons, neuron‐like electronics (NeuE) mimic neuronal components structurally and mechanically at the subcellular level [54]. In NeuE, round recording electrodes and long interconnects serve as probe building blocks that mimic the soma and neurites of a typical pyramidal neuron, while the insulation layer of interconnects mimics the myelin sheath (Figure 3c,d) [54]. By reducing feature dimensions to match the diameter of neurites, NeuE achieves bending stiffness comparable to neuronal axons [54]. The structural and mechanical similarity enables a seamlessly integrated interface between NeuE and neurons without noticeable disturbance [54].

NeuE mimics the soma, axon, and myelin sheath of neurons; however, it is designed for electrophysiology recording purposes and does not replicate the full functionality of a neuron. Future development of neuromorphic neurons should address both structural similarity and biological functionality.

### 3.3 Neuromorphic Neural Networks

Neurons in the brain are organized into layered, hierarchical architectures with three‐dimensional (3D) networks spanning multiple cortical layers. Different cortical layers exhibit distinct characteristics and functions; for example, superficial cortical layers have a higher synchronization frequency and greater spike‐field coherence than deeper layers [59]. Each layer contains different neuron types that form interlaminar connections: intratelencephalic (IT) neurons, found in layers 2–6, project axons within the telencephalon [60], while pyramidal tract (PT) neurons in layer 5 project to subcerebral targets including the brainstem, spinal cord, and midbrain and can also branch to the ipsilateral cortex, striatum, and thalamus [60].

To emulate these layered, hierarchical architectures, neuromorphic devices integrate individual elements into two‐dimensional (2D) layers or 3D structures [61]. Examples include memristor arrays with silicon CMOS circuits [62], and ferroelectric nanosynapse arrays (Figure 3e) [55]. The ferroelectric nanosynapse array exhibits learning behavior by tuning the conductances of nanosynapses (Figure 3f) [55]. Other efforts further integrate multiple 2D memristor arrays into complex neural networks, where the conductance of ferroelectric synapses can be mapped across different layers [63].

Hierarchical architectures mimic 2D layers and 3D structures of neural networks to some degree; however, biological neural networks are more complex. For example, the cortex is organized into six layers, with each cortical layer having distinct functions and connections. Future development of neuromorphic neural networks should include more biological components and complexity, such as projection neurons and interneurons.

## 4 Neuromorphic Devices Mimicking Neural Circuits

The body contains diverse peripheral receptors such as mechanoreceptors, photoreceptors, cochlear (acoustic) receptors, and gustatory receptors. The central nervous system receives and processes these inputs using ion channels, neurotransmitters, or proteins to modulate downstream responses. Together, sensing and modulation elements form functional circuits. Neuromorphic devices that emulate neural circuits target these circuit‐level behaviors. These devices translate external stimuli into electrical signals and incorporate modulation and feedback to recapitulate neural dynamics. Neural circuit‐mimicking neuromorphic devices integrate both functional elements and architectural organization to produce a closer correspondence to the biological nervous system. Different neuromorphic devices rely on distinct conversion principles and signal‐encoding methods, and therefore differ in power consumption and time delays (Table 2).

**TABLE 2:** Comparison of neuromorphic devices that mimic neural circuits.

Type	Pressure	Optical	Acoustic	Biochemical
Conversion principle	Mechanical deformation	Photon excitation	Mechanical vibration	Molecular binding
Signal‐encoding method	Spike frequency [64], postsynaptic current [64]	Event [65]	Spike frequency [66]	Conductance/voltage change [67]
Power consumption	∼pJ–fJ [68]	∼fJ [69]	Varies [70]	∼µW–mW [71]
Time delay	∼ms [72]	∼ms–s [73]	∼µs [66]	∼µs [71]

### 4.1 Pressure Sensing

Mechanical forces can deform cells or tissues and activate mechanically gated ion channels that transduce those forces into electrical signals [74]. Piezo2 is a mechanically activated ion channel expressed in sensory neurons of the dorsal root ganglion and Merkel cell‐neurite complexes [75]. The resulting ion flux is encoded into action potentials [76], and these signals are conveyed to the brain via the spinal cord dorsal columns [77].

**FIGURE 4:**
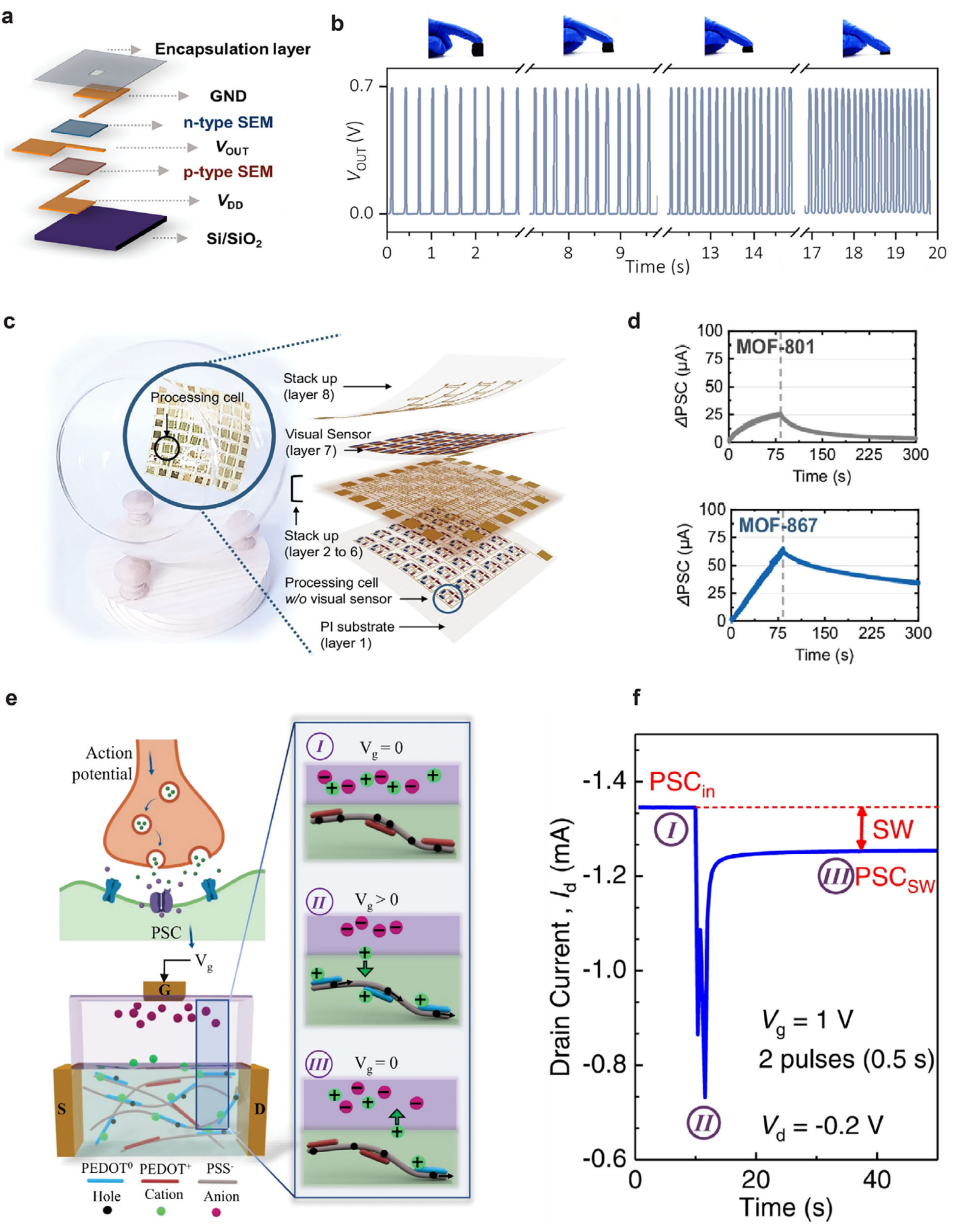
Neuromorphic devices mimicking neural circuits. (a), Exploded view of a complementary inverter mimicking mechanical perception. Produced with permission from [78], licensed under CC‐BY‐NC‐ND. (b), Voltage output vs. applied pressure for the device in a. Produced with permission from [78], copyright CC‐BY‐NC‐ND. (c), Schematic of a neuromorphic retina system. Reproduced with permission from [80], licensed under CC‐BY‐NC‐ND. (d), Response of the device from c shown as current vs. time. The two MOFs exhibit short‐term and long‐term plasticity, respectively. Reproduced with permission from [80], licensed under CC‐BY‐NC‐ND. (e), Schematic of a biological chemical synapse (top) and a neuromorphic chemical synapse (bottom). Reproduced with permission from [81], licensed under CC‐BY. (f), Postsynaptic current of the device in e vs. time. Reproduced with permission from [81], licensed under CC‐BY.

To emulate the mechanotransduction pathway, neuromorphic mechanical sensing systems couple pressure‐sensitive electronic components with spike‐generating circuits (Figure 4a) [78]. When external pressure is applied, the deformation of the polymer increases the effective contact area and thus reduces the device resistance (Figure 4b) [78]. For example, a pressure sensor is made by sandwiching a conductive polymer between two electrodes. The pressure‐dependent resistance change produces a corresponding voltage modulation analogous to the receptor potential in biological mechanoreceptors. A ring oscillator then receives the voltage output from the pressure sensor and converts this analog voltage into a train of voltage pulses that emulate neuronal action potentials. The oscillation frequency depends on the applied pressure, enabling rate coding in which stronger mechanical stimuli produce higher spike frequencies. A transistor‐based synaptic circuit then converts the spike trains into postsynaptic currents, completing a neuromorphic mechanosensory pathway from force detection to neural signal transmission [79].

Neuromorphic pressure sensing systems mimic biological pathways, including mechanoreceptors, afferent nerves, synapses, and central processing. To further improve pressure sensors, future development can focus on biological system integration, reducing power consumption, and higher scalability.

### 4.2 Optical Sensing

Rod and cone cells detect light in our eyes. Phototransduction occurs in the rods’ and cones’ outer segments, which contain the visual pigment rhodopsin [82]. Rhodopsins are G protein‐coupled receptors (GPCRs) that are activated by light and initiate the phototransduction cascade that underlies vision [83]. Graded potentials from rods and cones drive retinal ganglion cells (RGCs) to generate action potentials that travel to the brain [84].

To emulate this optical sensing pathway, neuromorphic devices integrate photodetectors with artificial synaptic and neuronal elements at the pixel level (Figure 4c) [80]. Photodetectors convert photons into analog electrical signals analogous to rod and cone responses [85, 86]. These neuromorphic optical systems can exhibit both short‐term and long‐term plasticity (Figure 4d) [80]. An artificial synapse in a transistor architecture converts the analog photodetector output into digital signals, which can then be transmitted to an external processor for post‐processing [80]. Weak or infrequent optical stimuli induce transient conductance changes corresponding to short‐term memory, whereas strong and repetitive stimuli stabilize synaptic conductance to form long‐term memory and learning [80]. One example of a light‐sensitive molecule is azobenzene. Incorporating azobenzene as the gate electrode and a PEDOT:PSS channel between source and drain yields a neuromorphic photoelectrochemical transistor [87]. Quantum dot‐based neuromorphic synapses can combine photodetection, memory, and neurostimulation on a single device [88]. Integrating learning and memory modules can further enhance neuromorphic visual performance.

Neuromorphic optical sensors convert photons into electrical signals with plasticity. However, the biological optical system includes multiple types of cells with distinct functions, and rods and cones possess different ON/OFF pathways. Future development of neuromorphic optical sensors can focus on better replicating the complexity of biological visual systems such as distinguishing light‐activated and dark‐activated responses, incorporating inhibition from other modules to mimic horizontal cells, and achieving direction selectivity.

### 4.3 Acoustic Sensing

The human ear contains three main elements: semicircular canal, saccule, and cochlea. Sound vibrations travel along the basilar membrane, where different regions resonate preferentially at different frequencies in the cochlea. Hair cells on the basilar membrane transduce these mechanical vibrations into electrical signals by opening mechanosensitive ion channels and releasing glutamate onto auditory nerve fibers that fire action potentials and convey auditory information to the brain [89].

To emulate acoustic sensing, neuromorphic circuits integrate mechanoelectrical transducers with frequency‐selective, and spike‐encoding circuits. MEMS microphones [66], piezoelectric devices [90], or triboelectric devices [91] convert sound‐induced vibrations into analog electrical signals [90]. In one representative example, researchers integrated piezo‐resistive deflection sensing with thermo‐mechanical actuation to mimic cochlear behavior [66]. Upon receiving sound, the acoustic sensors vibrate and generate electrical signals. Each sensor responds strongly to specific frequencies. In addition, integrating artificial synapses further enables learning and adaptation, as activity‐dependent plasticity can dynamically adjust frequency responses and support auditory learning at the sensor level [66].

Neuromorphic acoustic sensors transform vibrations into electrical signals. However, biological hearing is more than a cochlear filter bank; it is a closed‐loop adaptive neural system where the brainstem sends feedback to the cochlea. Future development of neuromorphic acoustic sensors can focus on integrating feedback mechanisms, binaural and spatial hearing to mimic two‐ear processing, and interfacing hardware systems with biological neural networks.

### 4.4 Biochemical Sensing

Taste and olfactory perception arise from chemosenzation. Taste receptor cells respond selectively to specific chemical compounds: binding of taste molecules activate distinct populations of receptors, modulate ion channels, and trigger neurotransmitter release onto gustatory neurons [92]. The combined activity of receptors encodes fundamental taste modalities such as sweet, umami, bitter, salty, and sour. The central nervous system (CNS) issues commands via efferent nerves that transmit signals to the peripheral nervous system (PNS). Propagation of action potentials conveys these commands by triggering ion fluxes, neurotransmitter release, and downstream mechanical or chemical responses such as muscle contraction and glandular secretion [93]. Similarly, olfactory sensory neurons (OSNs) sense odorants through GPCRs in the olfactory epithelium and transmit the resulting signals to the brain [94]. Signal transmission in the nervous system is primarily ionic: action potentials trigger localized ion fluxes and neurotransmitter release [93]. Proteins can complement ions in signal transmission: they carry net charges, permanent dipole moments, or heterogeneous surface charge distributions that, upon binding to specific receptors, locally modulate membrane potential and influence cellular responses [95]. Such protein‐mediated electrostatic modulation plays an important role in ion channel modulation and signaling [96].

To mimic chemoelectrical transduction, neuromorphic devices employ chemically selective materials that mimic taste or olfactory receptors. Ion‐selective electrodes [97], conducting polymers [98], metal–organic frameworks (MOFs) [99], and ion‐gels [81] selectively interact with target species such as H+, Na+, amino acids, and organic molecules [100]. Molecular binding or ion exchange at interfaces changes electrical properties such as conductance or potential [67], producing graded electrical signals analogous to biological receptor potentials. Neuromorphic ion‐modulation systems couple electrical signals with ionic dynamics in electronic materials: incoming spikes including voltage pulses, current transients, or frequency‐modulated waveforms drive ion redistribution within electrolyte‐gated transistors, ion‐gel capacitors, or memristive synapses (Figure 4e) [81]. The resulting ion motion modulates channel conductance or interfacial charge accumulation and translates into electrical outputs. Such devices can exhibit synaptic behaviors such as spike amplitude‐dependent plasticity (Figure 4f) [81]. Beyond ions, neuromorphic devices can emulate protein‐mediated signal transmission by incorporating biofunctionalized transistor gates that recognize target proteins. Protein binding alters the gate surface charge distribution and the local electrostatic environment, redistributing ions in the transistor channel. For example, in organic electrochemical transistors based on the conducting polymer PEDOT:PSS, ion motion modulates channel doping and conductivity and thus produces measurable changes in synaptic current [101].

Neuromorphic biochemical sensing systems bind to molecules and transform chemical signals to electric signals. However, in biochemical sensing systems, molecules are encoded by combinations of activated receptors. In addition, taste and olfactory systems are not isolated; they interact with other systems such as the gastrointestinal system. For example, ghrelin, produced primarily by the stomach, can modulate sniffing behavior and olfactory detection thresholds in rodents and humans [102]. Future development of neuromorphic biochemical sensing systems should focus on the combination of multiple sensors, formation of closed‐loop circuits, and integration with biological systems.

### 4.5 Integrating Sensing and Modulation

Biological neural circuits consist of afferent sensing pathways and efferent modulation pathways that together form closed‐loop control systems. Sensory receptors transduce mechanical, optical, acoustic, and chemical stimuli into electrical signals via the activation of voltage‐gated ion channels. Receptor potentials are integrated and, when the threshold is exceeded, action potentials are generated and transmitted to the brain via afferent nerves. In response, the brain then sends commands through efferent pathways to peripheral organs which produce responses.

To emulate these circuits, neuromorphic systems couple multimodal sensors with spike‐based processing and ionically mediated actuation. Mechanical, optical, acoustic, and chemical sensors convert external stimuli into analog electrical signals. For instance, a pressure sensor is used to form a neuromorphic sensorimotor loop [103]. This neuromorphic system includes multimodal reception, nerve‐like pulse‐train signal conditioning, and closed‐loop actuation [103]. Spatial and temporal processing modules, such as edge detection circuits, extract salient features analogous to sensory perception in the skin, and the resulting spikes are transmitted to the brain for further processing. Efferent neuromorphic pathways translate processed spike signals into modulation commands via synaptic transistors and neural interfaces [103]. The neuromorphic sensorimotor loop was connected to the rat somatosensory cortex to emulate skin sensation and trigger feedback responses in the motor cortex [103]. In the biological sensory feedback system, rats exhibited larger leg twitching angles in response to increased pressure inputs [103]. By integrating sensing, computation, and modulation within a unified framework, neuromorphic circuits realize a closed‐loop system that mirrors biological perception‐action systems [104].

The integration of a single sensing system with modulation has already been successful; however, sensory systems do not work in isolation. Future development should address the integration of multiple sensing systems as inputs to achieve more effective modulation.

## 5 Artificial Neuromorphic Computing

While advances in traditional von Neumann architecture had kept pace with Moore's law for decades, recent progress in computational power has slowed due to physical and architectural constraints in circuits using field‐effect transistors [105]. This has driven research into alternative architectures such as neuromorphic devices. Interfacing and controlling such hardware require new computing algorithms, and the rise of artificial intelligence (AI) and machine learning (ML) has further increased the demand for architectures that support powerful, large scale, parallel systems. Neuromorphic computing, which embeds online, real‐time updates with on‐processor memory provides a robust and efficient paradigm that exceeds traditional sequential instruction processing, and has garnered significant attention in the AI and ML fields.

Spiking neural networks (SNNs), sometimes described as the third generation of machine learning architectures, represent a fundamental paradigm shift from conventional artificial neural networks (ANNs) and are central to neuromorphic computing. SNNs aim to mimic the architecture and function of biological neural networks to achieve similar levels of robustness, adaptability, and efficiency [106]. SNNs are commonly implemented either as simulations on conventional von Neumann architecture or as hardware deployments on neuromorphic chips. Individual computational units in SNNs emulate the activity of neurons and send and receive discrete binary signals analogous to action potentials, as opposed to the continuous signals in ANNs. While ANNs integrate information by transmitting a weighted sum of inputs through a continuous, nonlinear activation function, SNNs transmit signals only once input exceeds a threshold. Unlike the highly connected and synchronous networks of ANNs, SNNs exhibit sparse connectivity and asynchronous, event‐driven activity. Construction of SNN models varies considerably depending on neuron models, learning, and encoding algorithms, and network architectures, but individual spiking units represent the most fundamental building block in SNN development.

### 5.1 Neuron Models

Neuroscientists have developed computational representations of neurons from experimental observations that form the foundation for understanding information processing in the CNS. Many mathematical models that approximate the stochastic behavior of neurons have been proposed and implemented in SNNs. These models are generally divided into two categories: (1) mathematical models that approximate the electrical activity of neurons, and (2) models that describe firing as a probabilistic product of stimulus inputs, i.e. neurotransmitter concentration or stimulus strength.

#### 5.1.1 Integrate‐and‐Fire Models

Electrical models have predominated in SNNs since they can be directly implemented on neuromorphic hardware. The simplest model, coined the Integrate‐and‐Fire (IF) model, was first conceptualized by Louis Lapicque in 1907. Lapicque studied the sciatic nerve of frogs and, using rudimentary electrical apparatus, related timed extracellular nerve stimulation and firing rate to an analog resistor‐capacitor circuit. This model described the electrical output of neurons as a function of the total stored membrane potential [107, 108]. While the IF model approximates neuronal function without the need to model underlying biological mechanisms, it has several problems, namely that memory of sub‐threshold currents is retained indefinitely, and neuron populations can phase lock under strong stimulations.

**FIGURE 5:**
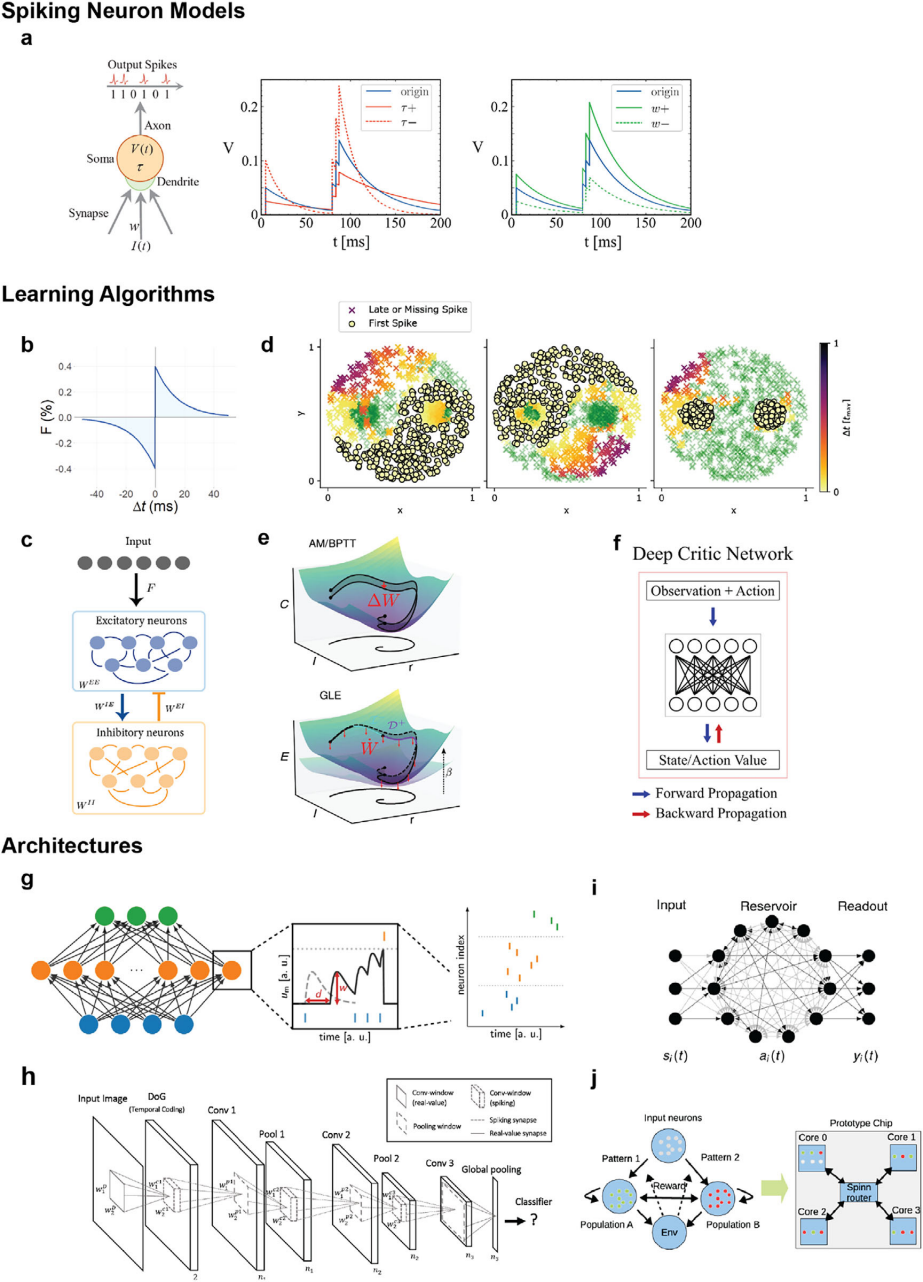
Examples of common spiking neuron models, learning algorithms, and architectures used in SNNs. (a), Schematic and functional response of the LIF neuron model with time and weight constants as implemented in PLIF. Reproduced with permission from [109], copyright IEEE Proceedings. (b), Spike timing‐dependent plasticity window. (c), Dual inhibitory‐excitatory neuron network architecture for dynamics optimization. Reproduced with permission from the authors of [110]. (d), Learning on the Yin‐Yang dataset using event‐based backpropagation. Reproduced with permission from [111], licensed under CC‐BY. (e), Gradient descent under the general latent equilibrium framework. Reproduced with permission from [112], licensed under CC‐BY 4.0. (f), Schematic of a deep actor‐critic network. Reproduced with permission from [113], licensed under CC‐BY 4.0. (g), Example feed‐forward SNN architecture. Reproduced with permission from [114], licensed under CC‐BY 4.0. (h), Example convolutional deep neural network. Reproduced with permission from [115], copyright Elsevier. (i), Schematic of a reservoir neural network. Reproduced with permission from [116], licensed under CC‐BY 4.0. (j), Schematic of the SpiNNaker2 prototype neuromorphic core. Reproduced with permission from [117], licensed under CC‐BY.

To address these issues, Knight introduced a ‘leaky’ current to the mathematical description of membrane potential, producing the Leaky Integrate‐and‐Fire (LIF) model which better recapitulates activity at the population level [118]. The LIF model remains a popular choice for implementation on simple analog neuromorphic devices [27, 30, 119, 128], memristors [38, 120], FGPAs [121], ASICs [122, 123], and for in silico spiking neural networks. The LIF model's single ordinary differential equation (ODE) is readily discretized into low‐level hardware and receiving, storing, and sending single‐bit spikes is readily adapted to existing addressing hardware or bus systems (Table 3). The model has been widely extended to describe the diverse activity patterns of neurons [144, 145, 146, 147]. While membrane constants such as resting potential or decay rate are often treated as network‐wide hyperparameters, researchers have recently introduced Parametric LIF (PLIF) to incorporate a membrane time constant as a learnable parameter, allowing for greater network heterogeneity, and demonstrating superior accuracy compared to standard LIF on image classification tasks using MNIST, CIFAR‐10, N‐MNIST, and DVS128 classification datasets (Figure 5a) [109]. Gated LIF (GLIF) combines learnable membrane constants with a gating mechanism to modulate their effect on LIF dynamics, though GLIF contains additional model overhead that makes it less suitable for large SNN models or Edge AI applications [148]. Recently, efforts have also been made to model adaptive behaviors observed in biological neurons [144, 145] by integrating dual leak mechanisms in LIF models. Two‐Compartment LIF (TC‐LIF) includes somatic and dendritic compartments to model short‐ and long‐term temporal adaptation, demonstrating superior convergence on temporal classification tasks compared to both PLIF and GLIF [149]. Dual Adaptive LIF (DA‐LIF) features only a single compartment, but introduces separate spatial and temporal constants to recapitulate adaptive firing [131]. Both TC‐LIF and DA‐LIF show improvements in computational efficiency and accuracy in computer vision tasks, such as on CIFAR10‐DVS and DVS128 gesture datasets [131, 149]. While all LIF variants introduce increased biological fidelity, GLIF, TC‐LIF, and PLIF have increased model overhead, reducing their energy efficiency and hardware compatibility compared to DA‐LIF. Importantly, to our knowledge, only GLIF has been implemented on neuromorphic hardware [150], which remains a major barrier for adoption of these advanced models. LIF models have also been used to explain the properties of biological neurons. For example, researchers at the Allen Brain Institute mapped the activity signatures of neocortical neurons in transgenic mice to generate LIF models of both general activity and genetic subpopulations [132], which could in turn inform future SNN development. While these advances in LIF models better recapitulate complex neuronal activity, models with a limited number of compartments still lack non‐linear properties, and further work introducing greater heterogeneity and compartments through cable theory is needed to capture the full richness of neural dynamics.

**TABLE 3:** Comparison of major neuron models used in spiking neural networks.

Neuron Models						
Model	Computational Cost	Parameters	Biological Fidelity	Power Efficiency	Hardware Implementability	Applications
LIF	1 ODE [118]	3‐4 [118]	Low	Very high	Analog and digital circuits [27, 30, 119] Memristor [38, 120] FGPAs [121] ASICs [122, 123]	Large SNNs [121] deep learning [124, 125, 126] edge AI [127, 128, 129] neuromorphic chips [123, 128, 130, 131] biological simulation [121, 132]
Izhikevich	2‐3 ODE [133]	4 [133]	Medium	High	FGPAs [134, 135] ASICs [123]	Medium SNNs [133, 134] neuromorphic chips [136] biological simulation [133]
Hodgkin‐Huxley	4+ ODE [137]	20+ [137, 138, 139]	High	Low	Memristors [27, 140] FGPAs [141] ASICs [123] GPUs [138, 139, 142, 143]	Biological simulation [138, 139, 142, 143]

#### 5.1.2 Hodgkin‐Huxley Models

Two decades prior to Knight's introduction of the LIF model, Hodgkin and Huxley developed a quantitative description of membrane currents from voltage‐clamp electrophysiological recordings of the squid giant axon. The model relates membrane potential to the movement of sodium and potassium ions through voltage‐gated channels and includes a description of leaky currents [137]. The Hodgkin‐Huxley model is highly biophysically accurate and captures detailed action potential shapes [151], but this biological accuracy comes at the cost of increased computational complexity, making the model not suitable for many neuromorphic applications (Table 3). Moreover, training networks composed of Hodgkin‐Huxley neurons is more difficult than other neuron models (Table 4). While previous work has applied Hodkin‐Huxley type models to neuristors [152], these representations only model selected descriptive equations of the original model. Several packages for biophysical simulations support the family of Hodgkin‐Huxley models and include extensions with additional ion channels, multiple compartments, and gap junctions [138, 142, 143]. The Hodgkin‐Huxley model has also been expanded to include multiple compartments that better describe morphological properties of neurons [141]. Hodgkin‐Huxley‐based simulations have been used to study the neocortex [153], motor cortex [154], and hippocampus [155]. Recent approaches have further optimized these simulation environments with GPU acceleration and parallelization [139], as well as implementations on field gate programmable arrays (FGPAs) [141], application‐specific integrated circuits (ASICs) [123], and limited implementations on neuromorphic memristive devices [140].

**TABLE 4:** Compatibility and training complexity of unsupervised and supervised learning algorithms with neuron models. ✔ denotes theoretical compatibility and ✖ denotes theoretical incompatibility. For each neuron model, the difficulty of training is approximated below using “Simple”, “Moderate”, and “Difficult” qualitative labels. * labels denote compatibility by using mathematical approximation techniques.

Unsupervised Learning Algorithm Compatibility						
Algorithm	LIF	Izhikevich	Hodgkin‐Huxley	Time‐stepped?	Event‐driven?	Online?
STDP	✔ Simple [115]	✔ Simple [135]	✔* Moderate [156]	✔	✔	✔
Supervised Learning Algorithm Compatibility						
Surrogate Gradients	✔ Simple [124, 131]	✔ Difficult [112]	✖ [139]	✔	✖	✖
Time‐based backprop	✔ Simple [109, 125, 157]	✔ Difficult [158]	✖	✔	✖	✖
Event‐based backprop	✔ Moderate [111]	✔ Difficult [159]	✖	✔*	✔	✖
Q‐learning	✔ Moderate [113, 160]	✔	✔ Difficult [161]	✔	✔	✔
Actor‐critic	✔ Moderate [162, 163]	✔ Moderate [162, 163]	✔ Moderate [164]	✔	✔	✔

#### 5.1.3 Ischevik Models

Izhikevich introduced a hybrid approach between simple LIF neurons and biophysical Hodgkin‐Huxley models. Izhikevich used bifurcation methodologies to distill the Hodgkin‐Huxley description into a system of ordinary differential equations that support fast and accurate modeling (Table 3) [133]. While less complex than Hodgkin‐Huxley‐based SNNs, Izhikevich neuron networks have increased training overhead compared to LIF model networks (Table 4). The Izhikevich model is widely adopted for biological simulations [138, 142] and implementation on neuromorphic devices, including FPGA‐based [134] and non‐volatile memory [136] systems. The model has also been extended for use with Cordinate Rotation DIgital Computer (CORDIC) algorithms, which provides a fast calculation method for the model's differential equations using only bit‐wise shifting, adding, and subtraction for efficient implementation on FPGA circuits [135].

### 5.2 Artificial Neuromorphic Learning

SNNs differ substantially from their conventional counterparts in their learning algorithms. Fundamentally, machine learning seeks to minimize the difference between predicted and actual targets by assigning credit across space or time to the individual computational units responsible. These methods take the form of unsupervised, supervised, or hybrid approaches. Neuromorphic learning draws on both neuroscience and machine learning theory, with algorithms inspired by ideas from either field. A central question in neuroscience is how biological neurons encode information; studies show substantial heterogeneity in neurons [165, 166], including rate encoding, temporal encoding, inter‐spike‐interval, and time‐to‐first spike encoding strategies [167, 168], though results across individual studies are sometimes conflicting [169]. Moreover, hardware implementation of these learning algorithms is another major factor when developing neuromorphic architecture. Developing robust and efficient learning algorithms for SNNs remains an ongoing challenge and an active area of research.

#### 5.2.1 Unsupervised Learning Methods

Unsupervised learning algorithms allow the network to understand latent patterns or relationships from unlabeled data. In neuromorphic computing, these rules are largely built from Hebbian theory [170], which enables neural networks to self‐organize based on input activity. Hebbian theory is often implemented in SNNs through spike‐timing‐dependent plasticity (STDP), which describes a cumulative strengthening mechanism when presynaptic and postsynaptic firing is causally correlated. STDP also describes competition among multiple presynaptic partners for control of postsynaptic timing, resulting in LTP or LTD of synaptic connections (Figure 5b) [171]. Similarly, spike‐rate‐dependent plasticity (SRDP) uses average pre‐ and post‐synaptic firing rates over longer timescales to inform synaptic connections [172].

A major challenge for Hebbian theory algorithms is runaway positive feedback. Neural networks avoid this through inhibitory neurons or non‑Hebbian mechanisms to maintain an excitatory/inhibitory (E/I) balance optimal for information processing [173, 174]. Inhibitory and excitatory properties have been implemented both within single neuron units using a cyclical STDP learning strategy (Figure 5c) [175], and as separate inhibitory units constructed in a minimax‐optimized architecture [110]. Homeostatic scaling achieves E/I balance by adjusting synaptic strengths in response to stimulation levels, maintaining relative synaptic weights, and controlling optimal network criticality [116]. Combining Hebbian learning and long‐term synaptic scaling has been demonstrated to support robust memory formation in SNNs [176]. STDP is considered the most widely used learning rule in neuromorphic computing for its simplicity (Table 4), with implementations both in virtual SNNs and directly on neuromorphic hardware. STDP can be implemented on memristive hardware, where the conductance of an artificial synapse can be tuned with voltage pulses [55, 177, 178]. Similarly, analog or digital CMOS circuits can be implemented to achieve on‐chip unsupervised learning. STDP can also be abstracted onto digital neuromorphic hardware, such as FGPAs, by tracking and updating weights using logic gates [135] and dynamic volatile memory [123, 128, 130]. SNNs using STDP have also shown robustness against adversarial attacks, likely due to the asynchronous nature of spiking signals [125, 179]. Other forms of synaptic plasticity observed in the brain include behavioral timescale synaptic plasticity (BTSP) in hippocampal CA1 pyramidal neurons, where strong inputs arriving seconds before or after complex network activity in spatial tasks produce place cells [180]. BTSP has been recently extended to produce an in silico one‐shot learning model of memory trace formation [181], which, to our knowledge, has not yet been implemented in neuromorphic devices or computing.

#### 5.2.2 Supervised Learning Methods

Supervised learning, in contrast to unsupervised methods, utilizes labeled data to train the network to learn specific input‐output pairings. In traditional ANNs, supervised learning is most commonly performed through gradient descent backpropagation. After a learning step, the gradient of the loss function is calculated and propagated back through the ANN, and the weights of each neuron are updated to minimize the loss. However, achieving supervised learning is challenging in SNNs, since the binary outputs of spiking networks are non‐differentiable, making the architecture not suitable for traditional backpropagation.

Backpropagation is therefore modified for use in SNNs either as surrogate gradients or as event‐ and time‐based propagation, with both techniques implemented as local and global learning strategies. While some studies have adapted existing backpropagation techniques through approximate or surrogate gradients [157], these approaches may fail to capture the precise timing of synaptic events during training. Other studies have focused on the event‐based or time‐delay exact backpropagation techniques. Event‐based backpropagation (EventProp) computes an error signal at the time of spiking for each neuron using voltage or spike timing loss functions, achieving performance competitive with traditional ANNs on the Yin‐Yang task (Figure 5d) [111]. EventProp has also been extended to include time delays in the loss function for recurrent SNNs [182]. Delay‐based gradients (DelGrad) implements explicit spike‐delay gradients without the need to track other variables, which is implemented directly on BrainScaleS‐2 for on‐chip learning [114]. More recently, the Generalized Latent Equilibrium (GLE) backpropagation method was developed, which utilizes dual time variables for past and future credit assignment (Figure 5e) [112]. While effective, backpropagation lacks a direct biological correlation, instead representing overall network‐level changes observed in natural learning processes [183]. Models using gradient‐ and backpropagation‐based techniques are more challenging to be trained than STDP approaches, though they show superior performance on precise classification tasks (Table 4). Both backpropagation and surrogate gradients have been implemented on memristive hardware [114, 184] and ASIC neuromorphic architectures [185, 186], though EventProp and GLE remain largely software simulations. Event‐ and time‐based backpropagation techniques represent a useful hybrid between machine learning algorithms and biological plausibility, enabling the use of existing devices and computing approaches to study SNNs.

Supervised learning in SNNs can also be accomplished with reinforcement learning (RL), which is a critical component of reward pathways in the brain involving dopamine and serotonin [187, 188, 189]. Learning in RL is achieved via trial‐and‐error, where the network learns how to maximize a reward function based on its actions in an external environment or task. Analogous learning signals have been implemented in SNNs using Q‐learning, which assigns an expected reward to actions taken by the SNN, and updates that value at each learning step to maximize reward [190]. The basic Q‐learning algorithm has also been extended for deep learning architectures (e.g. deep Q‐network, DQN), demonstrating efficient learning on high‐dimensional datasets [160]. Recently, this algorithm has been applied to spiking neurons using ANN‐to‐SNN conversion and implemented directly on neuromorphic hardware, showing improved learning in noisy environments [191]. Q‐learning has also been applied directly to in silico SNNs by implementing non‐spiking interneurons to store representations of learning values [192]. Similarly, reward‐modulated STDP has been used for RL, in which a temporally encoded SNN is reinforced by STDP on neurons that fired the earliest in a computer vision task [193].

RL in SNNs can also be implemented with actor‐critic networks, where action processing and evaluation are divided into separate networks. The actor determines the actions to execute according to the policy, and the critic evaluates those actions and improves the policy. Deep population‐encoding SNN implementations of actor‐critic networks have been shown to substantially improve representation capacity of the network (Figure 5f) [113], and actor‐critic SNNs perform well in spatial navigation tasks [162]. Actor‐critic networks model several key components of mammalian reward circuitry. Dopamine neurons in the ventral tegmental area (VTA) and substantia nigra pars compacta (SNc) integrate information about reward policies, and downstream striatal and cortical regions integrate this signal to drive behavior. The reward policy is then updated based on external reward, and integrated into internal representations via reward prediction error (RPE) signaling in the VTA [187, 194]. Actor‐critic networks, and by extension dopamine and RPE signaling, represent a third factor outside of basic Hebbian learning principles, enabling attribution of positive and negative values to actions. Dopamine signaling follows direct and indirect pathways via D1 and D2 receptors, which have predominantly excitatory or inhibitory effects on downstream regions [195]. At the hardware level, both Q‐learning and actor‐critic networks are compatible with memristive and digital neuromorphic devices [163, 191] (Table 4). Researchers have recently emulated Actor‐critic RL on memristive neuromorphic devices, demonstrating a fully local learning rule that recapitulates spatial navigation in task simulations [163].

### 5.3 Architectures and Applications of Neuromorphic Computing

For many years the accuracy of SNNs lagged behind large deep learning ANNs, but recent advances have brought SNNs on par with conventional ANN models. SNNs are arranged into networks of individual neurons to mimic common ANN architectures or brain connectivity. The most basic SNN implementations use a feed‐forward architecture, where neurons are arranged into layers that communicate unidirectionally (Figure 5g) [114, 193]. Feed‐forward architectures are commonly extended into convolutional and deep neural networks, which use locally connected layers, convolutional filtering layers, and pooling layers to process complex representations in image and video recognition tasks [124, 175, 196]. Feed‐forward and convolutional deep neural networks are broadly compatible with nearly all learning rules [112, 113, 114, 131, 148, 191]. STDP‐based deep convolutional SNNs have shown improvements over traditional convolutional ANNs (Figure 5h) [115]. SNNs can also be arranged into recurrent neural networks, where neuron layers share recurrent or backward connections with previous layers to store and recognize temporal dependencies in time‐series data [149, 196]. Recurrent neural networks can also be implemented as reservoir neural networks, where the reservoir is instantiated with random or pre‐defined weights, and the output layer is trained to interpret the inherent reservoir activity (Figure 5i) [116, 129].

Several industry‐led projects have produced devices that support multiple online learning and SNN implementations. Intel Labs created Loihi, a silicon implementation of LIF neurons connected by a mesh routing system that supports up to 131,000 neurons and 130 million synapses, enabling arbitrary network designs and offering 1000 times greater energy efficiency when training ML models using STDP [130]. IBM also developed their TrueNorth architecture, a custom CMOS neurosynaptic chip composed of many cores arranged in a 2D grid with interchip interfaces, totaling 1 million LIF neurons and 256 million synapses [122]. SpiNNaker, created by researchers at the University of Manchester, is a neuromorphic manycore processor composed of ASIC cores that emulate roughly 1000 neurons. Cores are in turn connected by spin routers to form a single chip, which is then interconnected to 1 million additional nodes to produce a supercomputer with nearly 1 billion individual model neurons [186]. A second generation of the architecture introduces efficiency improvements using hardware accelerators and per‐core power management, increasing the number of silicon neurons up to 5 billion (Figure 5j) [123]. SNNs especially excel at processing time series data due to the inherent inclusion of temporal processing in their architectures. Loihi has been used to implement a deep spiking neural network architecture with embedded temporal processing for electroencephalogram (EEG) analysis, demonstrating a 95% reduction in energy consumption compared to traditional deep neural networks [197]. Loihi has also been used to model the mammalian olfactory system for detection of odorant samples such as toluene and acetone, with potential applications in disease and hazardous substance sensing [198]. SNNs have also been applied to accelerate finite‐element analysis, which improved efficiency in otherwise computationally intensive scientific modeling [199]. More recently, SNN architectures have also been implemented for large language models (LLMs), including replacing transformer models with sequential spiking streams [126], and spiking bipolar neurons for efficient LLM implementations [200].

### 5.4 Scaling and Training Challenges

While large neuromorphic architectures and SNNs are generally more energy efficient than traditional ANNs, these models face several scaling challenges in training, synaptic weight storage, and communication overhead as network size increases. While SpiNNaker2 can support up to 5 billion neurons and has been used to demonstrate sophisticated structural plasticity and reward‐based learning models [117], this scale still represents only a small fraction of the estimated ∼90 billion neurons in the adult human brain [201]. Packets of information representing spiking activity need to be transmitted between neuromorphic cores at each time step. SpiNNaker2 utilizes a network‐on‐chip solution [123], whereas TrueNorth and Loihi use custom mesh grid structures to route spike traffic [122, 130]. Despite the complexity of routing communication traffic, memory access for retrieving synaptic weights remains a major bottleneck [202]. The SpiNNaker architecture partially addresses this issue by implementing a Direct Memory Access (DMA) controller in each core to speed up transfer from SDRAM to on‐chip memory [203]. Researchers have successfully performed real‐time simulations of 77 000 cortical neurons on SpiNNaker [204]; however this scale represents the practical boundary for real‐time communication. Recent efforts have sought to address scaling issues by reducing routing latencies through hierarchical routing [205] and improving synaptic weight storage via quantization [206]. However, further advances in both hardware architecture and algorithm design are needed to support larger SNNs.

SNNs also face challenges similar to traditional ANNs in the computation cost of training, as the energy efficiency advantages of SNNs are only ascertained during execution rather than training. Many SNNs implemented on neuromorphic and conventional hardware are first trained using GPUs prior to deployment. Training remains computationally intensive: for example, one study reported a time complexity of 144 s for just 200ms of simulations of 2000 Hodgkin‐Huxley model neurons on an Nvidia A100 GPU [139]. In studies examining SNNs for large language models, training times as long as 48 h for a single session have been reported [126, 200]. As such, there is significant interest in exploring on‐chip or fully local learning strategies. The GLE backpropagation method is fully online and local, allowing researchers to eliminate offline training entirely [112]. Both PLIF and DA‐LIF have demonstrated reduced training cost while maintaining comparable accuracy [109, 131]. Researchers have also demonstrated online learning on SpiNNaker2 to train recurrent SNNs in real time [207]. Advancing on‐chip and online learning methods for neuromorphic computing will continue to be an important research area to advance the scaling, efficiency, and accuracy of SNNs.

## 6 Biological Neuromorphic Computing

Recently, neuromorphic computing has embraced a new approach aimed at using living neurons as the computational substrate. Termed synthetic biological intelligence (SBI) [208] or organoid intelligence (OI) [209], this approach employs 2D or 3D neuron cultures interfaced with electrode arrays to provide input/output (I/O) with computer hardware. While recent advances have propelled this nascent field forward, future work is needed to achieve measurable learning or “intelligent” characteristics and address current challenges in interface design, neuron culture quality and consistency, and reproducibility in experimental findings.

### 6.1 2D Networks

Mammalian neural culture provides a controlled system to model the brain, enabling the study of neurons and their network development, connectivity, and function in health and disease. These neural cultures come either from primary neurons dissected from animals or from neurons derived from stem cells. To record real‐time activity, cultures can be imaged with calcium indicators or monitored for intra‐ or extracellular electrical activity by neural probes. Multielectrode arrays (MEAs) [210] have been essential tools for studying the electrophysiological activity of neural cultures, and recent developments in high‑channel‑count, high‑density arrays now enable precise mapping of biological neural network activity [211]. With these advances, MEAs are now used as interfaces with adherent in vitro neural cultures to generate fine‐grained, complex activity maps of neural cultures.

**FIGURE 6:**
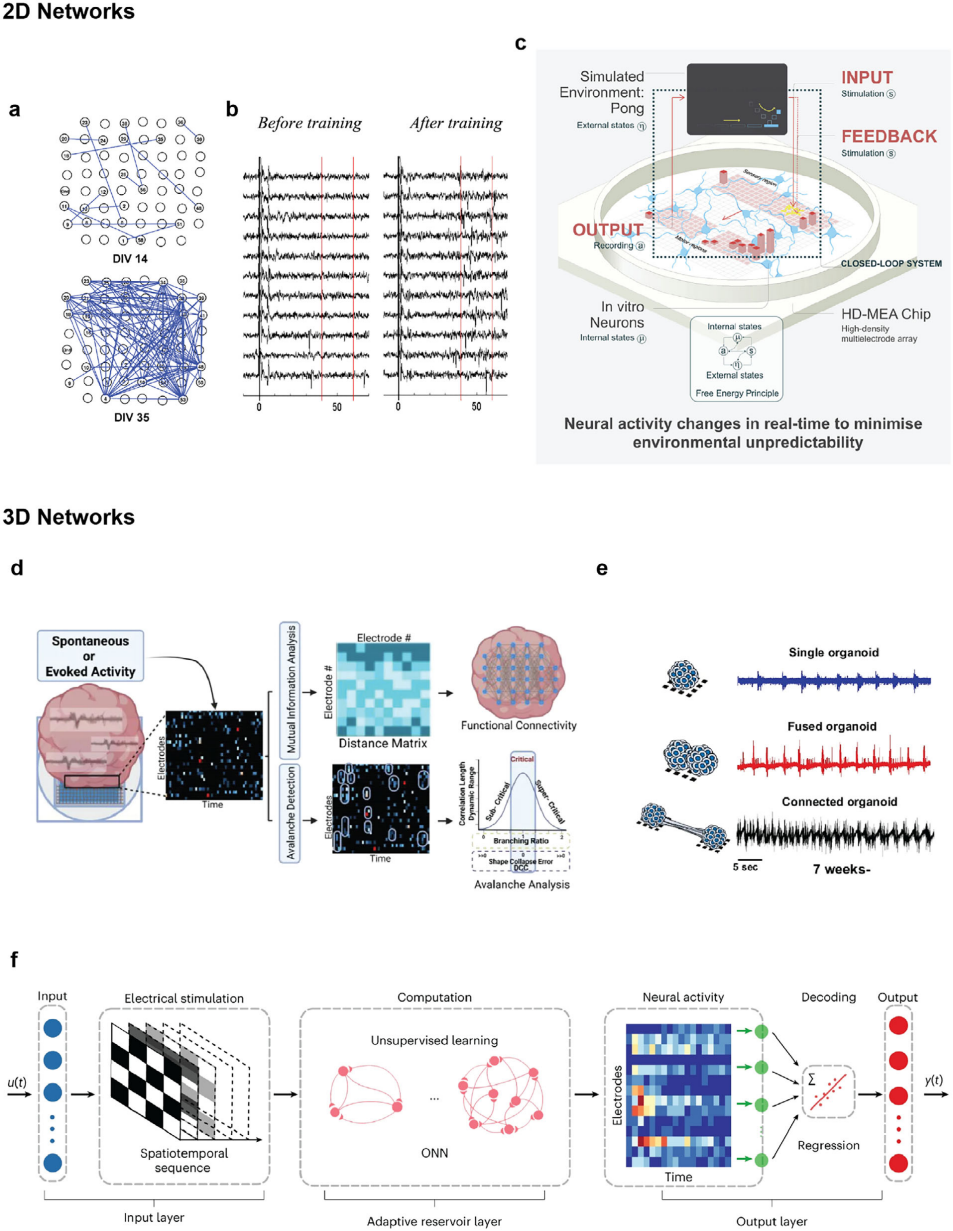
Examples of 2D and 3D biological neural networks for biohybrid computing. (a), Topological connectivity properties over time in adult rat cortical neurons plated on MEAs. Reproduced with permission from [215], licensed under CC‐BY. (b), Learning in rat cortical neurons following patterned electrical stimulation via an MEA. Reproduced with permission from [218], licensed under CC‐BY 4.0. (c), Diagram of the DishBrain system used to train neurons to play the game ‘Pong’. Reproduced with permission from [208], licensed under CC‐BY. (d), Schematic of functional connectivity and criticality in brain organoids. Reproduced with permission from [222], licensed under CC‐BY‐NC‐ND 4.0. (e), Electrophysiological recordings comparing signal complexity in single organoids, assembloids, and organoids connected by long‐range axonal projections. Reproduced with permission from [223], licensed under CC‐BY 4.0. (f), Schematic showing the use of cortical organoids as computational reservoirs. Reproduced with permission from [209], copyright Springer Nature.

In vitro biological neural networks exhibit key components necessary for basic learning and memory. Hippocampal neurons demonstrate functional connectivity [212] and short‐term and long‐term synaptic plasticity mediated by NMDA receptors [213, 214]. Mature cultures exhibit information‐processing optimized networks (Figure 6a) [215]. 2D neural cultures exhibit basic learning behaviors such as adaptation to patterned stimuli [216, 217] and entrainment of cortical bursting (Figure 6b) [218]. Recently, Cortical Labs demonstrated closed‐loop reinforcement learning with human and mouse induced pluripotent stem (iPS) cell‐derived cortical neurons that learned to play the game “Pong” via electrical stimulation using high‐density MEAs (Figure 6c) [208]. Reinforcement signals were delivered using a free energy minimization framework through coherent and incoherent electrical stimulation [208]. This work received significant media and scholarly attention and sparked discussion regarding the interpretation of the observed behaviors [219]. Subsequent studies using electrically hysteretic hydrogels have reported similar levels of learning in ‘Pong’ [220]. These works represent an important step toward using in vitro biological neural networks to produce pseudo‐cognitive responses. The discussion around the interpretation of these findings has highlighted the need for more consistent cross‑disciplinary definitions of computational processing, memory, and cognition. The question of what constitutes intelligence and/or sentience remains an open one [221], and additional research into the biological and mechanistic bases of intelligence can provide valuable insight.

### 6.2 Three‐Dimensional Networks

iPS cells can also be assembled into 3D aggregates, which are differentiated into 3D organoids that recapitulate key aspects of neural development and disease, including cytoarchitecture and functional activity [224, 225]. These differentiation approaches have been used extensively to produce either unguided or regionalized neural organoids [226, 227]. Organoids contain neurons that form synaptic connections and exhibit functional activity [227] as well as the machinery necessary for neural communication, including inhibitory and excitatory receptors and expression of genes associated with synaptic plasticity (Figure 6d) [222]. At the network level, studies have reported oscillatory dynamics [228] and structured firing patterns [229] in organoids and assembloids (Figure 6e) [223], demonstrating their utility as models for studying neural circuitry.

From a computational perspective, a key advantage of 3D organoid models over monolayer cultures is their greater non‐linearity, which provides a higher‐dimensional space for complex network communication. This complexity is further enhanced by longer culture periods and the inclusion of supporting cells such as astrocytes and oligodendrocytes. Recent work has used cortical organoids as computational reservoirs, where a high‐dimensional, fixed‐weight network (reservoir) receives inputs, and a readout function is trained to map the intrinsic activity of the reservoir to outputs or data labels (Figure 6f) [209]. In this study, 2D MEAs provided the electrical interface to encode time series input as spatiotemporal electrical stimulations. Cortical organoids successfully predicted nonlinear chaotic equations and classified speech in a recognition task with less training data than in silico ANNs [209]. Organoids undergoing training also showed functional connectivity changes and adaptation to input data, further demonstrating their potential as computational units [209]. While this work represents a key step toward utilizing brain organoids as computational units, further work is needed to validate both the intrinsic computational power of organoids and their robustness as reservoirs. Computational reservoirs require properties such as stable internal networks, fading memory, input separability, and nonlinearity. While some reservoir properties have been demonstrated [209], standard quantitative benchmarks for reservoir capabilities such as memory capacity tests [230], kernel rank analysis [231] for nonlinear separability, and time‐series tasks such as NARMA or Mackey‐Glass remain to be tested on brain organoids. Learning in reservoir computing paradigms is handled by an in silico readout layer rather than by the reservoir itself, and even basic physical systems as simple as a bucket of water have been shown to exhibit limited learning abilities [117]. Nevertheless, reservoir approaches offer useful computational power even with relatively simple properties, and offer a practical and scalable route to harness complex biological dynamics for learning.

Using human‐derived tissue models, especially for computational or biohybrid applications, raises a number of ethical concerns regarding consciousness, sentience, and donor consent. Current brain organoid models are generally considered to be non‐conscious [232, 233], as they lack structural maturity, sensory abilities, and complex brain circuitry. However, continued refinement of culturing parameters [228] and research into more complex assembloids [223, 226] raises the possibility that organoids may gain limited sentience. Particularly, the definitions of sentience and consciousness are open to debate and interpretation [221], and detecting emergent consciousness is challenging. Using iPS cell‐derived tissues also carries additional ethical challenges related to donor consent. Patients are legally and ethically entitled to informed consent, but may not be aware of the potential for their donated cells to result in cognitive tissues. Researchers have also raised concerns regarding the distinction between ‘subject‐like’ and ‘object‐like’ properties of organoids, and how this distinction changes the ethical framework used for evaluating these models [234]. While the present likelihood of the emergence of sentient or conscious properties in organoids is low, researchers must consider developing ethical standards and definitions to balance ethical responsibility with scientific innovation.

Work remains to evaluate the potential of using organoids for more complex learning and cognition tasks, including addressing variability in organoid cultures and challenges in interface technologies. Learning through molecular synaptic plasticity and internal representation formation in organoids during an intelligence task has not yet been demonstrated and remains an important open question. Moreover, organoid maturity and functional quality depend heavily on the source and handling of iPS cells, resulting in variability [226]. For electrical interfaces, while planar MEAs are suitable for monolayer cultures, the mismatch between planar MEAs and 3D organoids provides a less stable recording platform, and can introduce mechanical stress [235] and reduced physiological fidelity. Recent advances in flexible shell, buckled, and embeddable bioelectronic interfaces for organoids [235, 236] have the potential to improve biocompatibility and enable long‐term tracking and modulation of neural activity, providing a path toward more complex learning‐based tasks. If these challenges are addressed, the potential for organoid‐based biohybrid computing is immense. Modeling pseudo‐cognitive responses in human‐derived tissue would allow researchers to study neurodevelopmental diseases with greater fidelity, accelerating our understanding of human diseases and treatments.

## 7 Summary and Future Outlook

Von Neumann computing limits further development of conventional devices due to the separation of computation and memory, which drives researchers to explore a variety of neuromorphic devices to mimic neuronal functions. MOSFETs can mimic soma‐like integration and threshold behavior. Memristors and electrolyte‐gated transistors can mimic the integration, propagation, and summation functions of neuronal dendrites. Memristors and devices based on PCM or ferroelectric materials can emulate synaptic plasticity. Integrating individual neuromorphic devices enables the emulation of more complex neural architectures: multi‐gated MOSFETs mimic dendritic trees, neuron‐like electronics mimic neurons, and 2D arrays and 3D assemblies of individual devices mimic neural networks. Neuromorphic devices have also been designed to mimic neural circuits for pressure, optical, acoustic, and biochemical sensing, as well as to implement integrated closed‐loop neural circuit functions.

While neuromorphic devices offer important advantages such as nonvolatility of mimicking short‐term plasticity, low energy consumption, and high scalability, there are still several issues that need to be addressed. First, variability exists among the same kind of neuromorphic devices, including filamentary switching variability [38], cycle‐to‐cycle variability, device‐to‐device variability, and state‐retention drift [46]. Second, current neuromorphic devices fail to replicate both neuron morphology and functions at the same time. Third, neuromorphic neural circuits are still working in isolation. To address these issues, future development of neuromorphic devices should focus on stability during fabrication and use, simultaneous replication of complex neural functions and architectures, and integration of different neuromorphic systems as well as real biological systems.

While conventional architectures have long relied on abstraction between devices and computing for ease of development, future systems will require tighter integration. Next‐generation neuromorphic devices and computing seek to model the adaptive, lifelong learning of biological neurons. Traditional ANNs suffer catastrophic forgetting under context shifts, whereas biological networks are highly adaptive and noise‐robust. Chip‐and‐organoid‐in‐the‐loop paradigms will combine on‐device learning with biomimetic devices, blurring the boundary between biological and physical components. Future neuromorphic systems will integrate devices and computing algorithms with online learning within a single chip for more robust and adaptable learning and sensation.

Several milestones need to be met before neuromorphic and biohybrid computing can achieve these goals. For neuromorphic computing, challenges with training efficiency and accuracy, synaptic weight updates, and communication overhead limit the accuracy of both biological simulations and task‐oriented SNNs. The authors expect that advances in learning algorithm design will allow SNNs to surpass traditional ANNs in accuracy, and that further development of neuromorphic chips will allow the realization of energy‐efficient and biologically accurate SNNs at scale. Major milestones will further include bringing per‐synaptic event efficiency below a femtojoule, which is on par with the brain, and 3D integration of neuromorphic technologies to model complex networks. For biohybrid computing, current limitations include interface design, tissue complexity and longevity, and training methods. While high‐density or embeddable microelectrode arrays are now used in neuroscience research, many have limitations including tissue deformation, limited stimulation capacity, or low spatial resolution precision. We expect future research to enable chronically stable, high‐resolution interfaces allowing biodirectional communication on up to thousands of channels between computers and biological tissue. Current 3D cultures contain diverse cell types, but lack the full cellular composition and organization needed to build complex neural circuits. To address this limitation, advanced tissue models such as assemboids or connectoids will be leveraged to model more complex brain circuitry. Finally, current biohybrid computing approaches are limited by learning methods and encoding strategies. Future advances in biohybrid computing will require leveraging more than rate‐ or intensity‐coded electrical stimulations; utilizing precise neurotransmitter release or optogenetic stimulation of neuronal sub‐populations to tune network activity will be necessary for more advanced learning tasks.

The future of neuromorphic devices and computing should integrate function, architecture, and circuit mimicry to create more sophisticated systems. Future efforts also need to address the growing computational demands of AI. Biological environmental factors such as the local microenvironment, extracellular matrix, and supporting cell types like astrocytes, oligodendrocytes, and microglia should also be taken into consideration. We envision a unified, bi‐directional approach: neuromorphic devices will be designed to more closely mimic biological neurons, and biological neurons will be harnessed through biohybrid computing to interface with conventional or neuromorphic devices. Seamless integration of biological and manmade systems promises to accelerate and deepen our understanding of how neurons store, process, and transmit information.

## Author Contributions

Z.Z., N.S., and X.Y. contributed to the conceptualization, writing, and revision of this manuscript.

## Conflicts of Interest

The authors declare no conflicts of interest.

## Data Availability

Data sharing not applicable to this article as no datasets were generated or analysed during the current study.
